# Conjugar Tecnologia com Qualidade na Assistência Médica

**DOI:** 10.36660/abc.20240127

**Published:** 2024-04-09

**Authors:** Luiz Maurino Abreu

**Affiliations:** 1 Hospital Federal dos Servidores do Estado – Cardiologia Rio de Janeiro RJ Brasil Hospital Federal dos Servidores do Estado – Cardiologia, Rio de Janeiro, RJ – Brasil; 2 Estimulocor – Aval Clinica e Cardiológica Rio de Janeiro RJ Brasil Estimulocor – Aval Clinica e Cardiológica, Rio de Janeiro, RJ – Brasil

**Keywords:** Assistência Médica, Assistência Primária de Saúde, Doenças Cardiovasculares, Telemedicina, Serviços Básicos de Saúde

Primeiro o RX, e depois o eletrocardiograma, revolucionaram a abordagem diagnóstica na cardiologia. Aparelhos médicos se sofisticam a cada dia, com o uso cada vez maior de ferramentas diagnósticas, agora dispondo da possibilidade de transmissão à distância dos exames, antecipando diagnóstico, e permitindo a aplicação de protocolos de atendimento e de decisão terapêutica.

Na cardiologia, com a transmissão do traçado eletrocardiográfico para centros cardiológicos em tempo real, o que a princípio era feito através de aparelhos de fac-símile, e depois, com dispositivos com transmissão pela internet, foi possível a adoção de protocolos de tratamento, com impacto na letalidade e no prognóstico funcional. Programas de teleconsultoria no contexto do infarto agudo do miocárdio, com transmissão de eletrocardiograma feito no primeiro local de atendimento, com este fim, foram desenvolvidos no Rio de Janeiro,^1^ (Projeto TIET), Salvador,^2^ São Paulo^3^ e Minas Gerais^4^ (Minas Telecardio), todos dentro do Sistema Único de Saúde (SUS), onde ficou demonstrado a segurança e o benefício de utilizar a teleconsultoria, abreviando o tempo para realizar o método de revascularização mais adequado.

Neste número dos ABC Cardiol, temos os dados de um protocolo piloto dentro do Estudo PROVAR+, realizado dentro da mesma Rede Minas, no qual o objetivo dos autores foi avaliar a acurácia da Tele-ECG em locais distantes para prever anormalidades no ecocardiograma de triagem, realizados por profissionais de saúde não médicos em Centros de Atenção Primária com laudo à distância por especialistas.^5^ Apesar das limitações citadas no próprio artigo, anormalidades no Tele-ECG aumentaram a probabilidade de doença cardíaca importante na ecografia de triagem, gerando boas perspectivas em agilizar o processo de estratificação e regulação no cenário da atenção primária.

A telemedicina tem grande potencial como fator de transformação da rede de assistência à saúde, inclusive na atenção primária. Este modelo de prestação de cuidados de saúde à distância oferece uma série de benefícios e desafios que devem ser cuidadosamente avaliados, um problema não só do Brasil, mas presente em diversos outros países do mundo.^6 , 7^

Mesmo com projetos em andamento, há barreiras. O artigo de Damasceno et al.,^8^ de 2019, com dados obtidos de questionário com 385 médicos que atuavam na Estratégia de Saúde da Família (ESF) em 73 municípios no Norte do Estado de Minas Gerais, quanto à utilização da Teleconsultoria ofertada pela citada Rede de Teleassistência de Minas Gerais que desenvolve o PROVAR+, mostrou que 55,8% dos médicos não a utilizavam, sendo identificadas como razões, desde a não disponibilidade de computadores e acesso à Internet nas Unidades Básicas de Saúde até a falta de informação e de formação em consultoria em telessaúde. Os resultados demonstram o grande desafio de implementação deste tipo de programa em todo o país e todo mérito de quem a desenvolve.

Há ainda um grande entrave para o Sistema de Saúde, que é o processo de regulação após o diagnóstico inicial, quando se teria de garantir equidade ao acesso à assistência médica de qualidade. Os Sistemas de Regulação não contemplam as necessidades de saúde da população. São grandes filas de espera, com impacto direto na prevenção e sucesso de tratamento.^9^ Novamente, isso não é exclusivo do Brasil e há preocupação da própria Organização Mundial da Saúde com o represamento de pacientes na Atenção Primária, impedindo o objetivo da proposta atual da Cobertura Universal da Saúde.^10^

Embora a telemedicina possa vir a representar um acesso mais amplo aos cuidados de saúde, especialmente para populações em áreas remotas ou com dificuldades para atendimento em centros médicos tradicionais, existem desafios a serem superados. Para maximizar os benefícios da telemedicina na atenção primária, é essencial integrá-la de forma holística aos sistemas de saúde existentes. Isso inclui a formação de profissionais de saúde para a utilização eficaz das tecnologias de telemedicina, o desenvolvimento de diretrizes e padrões de prática clínica específicos para consultas remotas, e a garantia de que a infraestrutura tecnológica seja robusta o suficiente para suportar a demanda crescente por serviços de saúde digitalizados. O crescimento tecnológico é extremamente rápido, com a perspectiva de a Inteligência Artificial ocupar boa parte do processo de estratificação de risco e diagnóstico, com potencial de revolucionar os Sistemas de Saúde, mas sua implementação plena vai exigir extensa regulamentação legal e ética.^11^

Em resumo, a telemedicina tem grande potencial dentro dos diversos níveis de atenção à saúde, ao permitir acesso mais amplo e eficiente para o diagnóstico e o tratamento. No entanto, para que isso seja alcançado com sucesso, é crucial abordar os desafios e garantir uma implementação cuidadosa e integrada dentro dos sistemas de saúde existentes, com financiamento adequado e a fundamental integração multiprofissional, que já ocorre em vários países desenvolvidos com legislação não restritiva. Sem isso será mais uma boa ideia, perdida em meio a burocracia e ao desperdício. Os dados apresentados nesta etapa do PROVAR+, são promissores e merecem atenção.
