# Supplementary material for: Conjugar Tecnologia com Qualidade na Assistência Médica
Source: Arq Bras Cardiol. 2024 Apr 9;121(2):e20240127. [Article in Portuguese] doi: 10.36660/abc.20240127 (PMC11081125; doi:10.36660/abc.20240127)
Supplement: Translation

# Translation: Combining Technology with Quality in Medical Care

**Authors:** Luiz Maurino Abreu

**Affiliations:** 1 Rio de Janeiro RJ Brazil Hospital Federal dos Servidores do Estado – Cardiologia, Rio de Janeiro, RJ – Brazil; 2 Rio de Janeiro RJ Brazil Estimulocor – Aval Clinica e Cardiológica, Rio de Janeiro, RJ – Brazil

**Keywords:** Medical Assistance, Primary Health Care, Cardiovascular Diseases, Telemedicine, Basic Health Services

First, the X-ray, and then the electrocardiogram, revolutionized the diagnostic approach in cardiology. Medical devices become more sophisticated every day, with the increasing use of diagnostic tools, now offering the possibility of remote transmission of exams, anticipating diagnosis, and allowing the application of care protocols and therapeutic decisions.

In cardiology, with the transmission of the electrocardiographic tracing to Cardiological Centers in real-time, which at first was done through facsimile devices, and later, with devices with internet transmission, it was possible to adopt treatment protocols, with impact on lethality and function prognosis. Teleconsulting programs in the context of acute myocardial infarction, with transmission of an electrocardiogram performed at the first place of care, for this purpose, were developed in Rio de Janeiro^1^(TIET Project), Salvador,^2^São Paulo^3^and Minas Gerais^4^(Minas Telecardio), all within the public health system (Sistema Único de Saúde - SUS), where the safety and benefit of using Teleconsultation was demonstrated, shortening the time to perform the most appropriate revascularization method.

In this issue of ABC Cardiol, we have data from a pilot protocol within the PROVAR+ Study, carried out within the same Rede Minas, in which the authors’ objective was to evaluate the accuracy of Tele-ECG in distant locations to predict abnormalities in the screening echocardiogram, carried out by non-medical health professionals in Primary Care Centers with remote reports by specialists.^5^Despite the limitations mentioned in the article itself, abnormalities in the Tele-ECG increased the probability of significant Heart Disease on screening ultrasound, generating good prospects for streamlining the stratification and regulation process in the primary care setting.

Telemedicine has great potential as a factor in transforming the health care network, including primary care. This model of providing healthcare remotely offers a series of benefits and challenges that must be carefully evaluated, a problem not only in Brazil but present in several other countries around the world.^6 , 7^

Even with projects underway, there are barriers. The article by Damasceno et al.^8^from 2019, with data obtained from a questionnaire with 385 doctors who worked in the Family Health Strategy (ESF) in 73 municipalities in the North of the State of Minas Gerais, regarding the use of Teleconsulting offered by the aforementioned Health Network Teleassistance in Minas Gerais, which develops PROVAR+, showed that 55.8% of doctors did not use it, with reasons being identified as ranging from the non-availability of computers and Internet access in Basic Health Units to the lack of information and training in telehealth consultancy. The results demonstrate the great challenge of implementing this type of program across the country and all the merit of those who develop it.

There is still a major obstacle for the Health System, which is the regulation process after the initial diagnosis when it would be necessary to guarantee equal access to quality medical care. Regulatory Systems do not address the health needs of the population. There are long waiting lists, with a direct impact on prevention and treatment success.^9^Again, this is not exclusive to Brazil and there is concern from the World Health Organization itself about the backlog of patients in Primary Care, impeding the objective of the current proposal for Universal Health Coverage.^10^

Although telemedicine may represent broader access to healthcare, especially for populations in remote areas or those with difficulty accessing traditional medical centers, there are challenges to be overcome. To maximize the benefits of telemedicine in primary care, it is essential to integrate it holistically into existing healthcare systems. This includes training healthcare professionals to effectively utilize telemedicine technologies, developing specific clinical practice guidelines and standards for remote consultations, and ensuring that technological infrastructure is robust enough to support the growing demand for services. digitized healthcare. Technological growth is extremely rapid, with the prospect of Artificial Intelligence occupying a large part of the risk stratification and diagnosis process, with the potential to revolutionize Health Systems, but its full implementation will require extensive legal and ethical regulation.^11^

In summary, telemedicine has great potential within the different levels of health care, by allowing broader and more efficient access to diagnosis and treatment. However, for this to be achieved successfully, it is crucial to address the challenges and ensure careful and integrated implementation within existing health systems, with adequate financing and the fundamental multidisciplinary integration, which already occurs in several developed countries with non-restrictive legislation. Without this, it will be another good idea, lost amid bureaucracy and waste. The data presented in this stage of PROVAR+ are promising and deserve attention.
